# The Octopamine Receptor OAMB Mediates Ovulation via Ca^2+^/Calmodulin-Dependent Protein Kinase II in the *Drosophila* Oviduct Epithelium

**DOI:** 10.1371/journal.pone.0004716

**Published:** 2009-03-05

**Authors:** Hyun-Gwan Lee, Suman Rohila, Kyung-An Han

**Affiliations:** 1 The Huck Institute Genetics Graduate Program, Pennsylvania State University, University Park, Pennsylvania, United States of America; 2 Department of Biology, Pennsylvania State University, University Park, Pennsylvania, United States of America; Center for Genomic Regulation, Spain

## Abstract

Ovulation is an essential physiological process in sexual reproduction; however, the underlying cellular mechanisms are poorly understood. We have previously shown that OAMB, a *Drosophila* G-protein-coupled receptor for octopamine (the insect counterpart of mammalian norepinephrine), is required for ovulation induced upon mating. OAMB is expressed in the nervous and reproductive systems and has two isoforms (OAMB-AS and OAMB-K3) with distinct capacities to increase intracellular Ca^2+^ or intracellular Ca^2+^ and cAMP *in vitro*. Here, we investigated tissue specificity and intracellular signals required for OAMB's function in ovulation. Restricted OAMB expression in the adult oviduct epithelium, but not the nervous system, reinstated ovulation in *oamb* mutant females, in which either OAMB isoform was sufficient for the rescue. Consistently, strong immunoreactivities for both isoforms were observed in the wild-type oviduct epithelium. To delineate the cellular mechanism by which OAMB regulates ovulation, we explored protein kinases functionally interacting with OAMB by employing a new GAL4 driver with restricted expression in the oviduct epithelium. Conditional inhibition of Ca^2+^/Calmodulin-dependent protein kinase II (CaMKII), but not protein kinase A or C, in the oviduct epithelium inhibited ovulation. Moreover, constitutively active CaMKII, but not protein kinase A, expressed only in the adult oviduct epithelium fully rescued the *oamb* female's phenotype, demonstrating CaMKII as a major downstream molecule conveying the OAMB's ovulation signal. This is consistent with the ability of both OAMB isoforms, whose common intracellular signal *in vitro* is Ca^2+^, to reinstate ovulation in *oamb* females. These observations reveal the critical roles of the oviduct epithelium and its cellular components OAMB and CaMKII in ovulation. It is conceivable that the OAMB-mediated cellular activities stimulated upon mating are crucial for secretory activities suitable for egg transfer from the ovary to the uterus.

## Introduction

Mating activates highly coordinated physiological processes in the *Drosophila* female. During copulation, the female receives somatosensory stimulation, sperm and seminal proteins from the male. These mating signals act at multiple sites in the mated female to activate post-mating responses required for successful reproduction [1], [2]. For example, the seminal protein Ovulin stimulates egg-laying for 1 day after mating [3]. Ovulin is not only present in the base of the ovary right after copulation but it also enters the circulatory system, possibly acting at additional sites [4], [5]. Moreover, the seminal sex peptides Acp70A and DUP99B reduce sexual receptivity and stimulate egg-laying [6]. While sex peptides have widespread binding sites in the central nervous system, endocrine glands, and reproductive tissues in the female [7], it is the sex peptide receptor SPR in the neurons expressing the sex determination factor Fruitless that is indispensable for reduced receptivity as well as increased egg-laying [8].

The downstream targets and mechanisms that Ovulin and SPR activate in the mated female are unknown. Several studies, nonetheless, indicate octopamine as a key neuromessenger for ovulation [9]–[13], suggesting it being a downstream signal of Ovulin or SPR for egg-laying. Octopamine is a major monoamine in insects and has similar functions to mammalian norepinephrine. Octopamine is synthesized from tyrosine by sequential actions of tyrosine decarboxylase (dTdc) and tyramine beta-hydroxylase (Tβh). The females defective in d*Tdc2* encoding neuronal dTdc or *tβh* are sterile due to defective egg-laying [10], [11]. Octopaminergic neurons innervate numerous brain and thoracico-abdominal ganglion (TAG) areas [14], [15]. In addition, octopaminergic neurons in the TAG project to reproductive tissues such as the ovaries, oviducts, sperm storage organs and uterus [12], [13]. Indeed, the sterility of *tβh* females is rescued by restored Tβh expression in a subset of neurons including the TAG neurons that innervate the reproductive system [10]. Consistently, octopamine, when applied to the dissected reproductive system, modulates muscle activities in a tissue-specific manner: it enhances muscle contraction in the ovary but inhibits it in the oviduct [12], [13]. This suggests that distinct octopamine receptors present in the ovary and oviduct mediate the opposite actions of octopamine on muscle activity.

*Drosophila* has four known octopamine receptors: OAMB, Octβ1R, Octβ2R and Octβ3R [16]–[18]. The *oamb* gene encodes two isoforms OAMB-K3 (K3) and OAMB-AS (AS), which are produced by alternative splicing of the last exon, and differ in the third cytoplasmic loop and downstream sequence. Both K3 and AS transcripts are found in the brain, TAG and reproductive system [9], [16]. When assayed in the heterologous cell lines, both isoforms activate an increase in intracellular Ca^2+^ [16], [18] while K3 also stimulates a cAMP increase [16]. This implies that the two isoforms may activate distinct combinations of signal transduction pathways *in vivo*. To investigate OAMB's *in vivo* functions, we have previously generated several *oamb* mutants defective in both K3 and AS and their prominent phenotype is female sterility [9]. While *oamb* mutant females show normal mating, they are impaired in ovulation, causing abnormal retention of mature eggs in the ovary [9]. This raises several important questions regarding mechanism of OAMB activity: where (brain, TAG or reproductive system) does OAMB regulate ovulation? Which isoform is critical for this process and what are the downstream signals? In this report, we show that the critical site for the OAMB's function in ovulation is the oviduct epithelium, in which transgenic expression of either K3 or AS isoform is sufficient to rescue the *oamb* female's ovulation defect. Moreover, we also demonstrate that OAMB recruits CaMKII as a key downstream effector for this function.

## Results

### Either OAMB isoform is sufficient to rescue defective ovulation in oamb females

**Figure 1:**
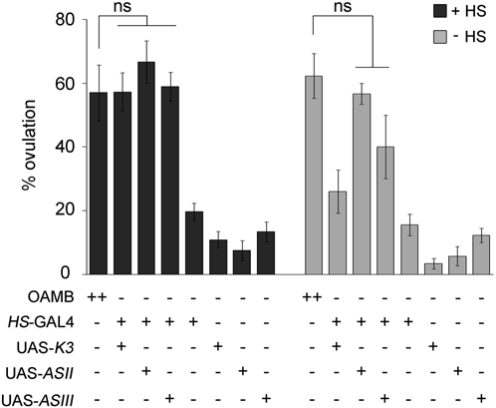
Rescue of *oamb*'s impaired ovulation by ubiquitous expression of individual OAMB isoforms. Flies reared at 18°C were treated with heat shock (+HS) to induce HS-GAL4 expression or maintained at 18°C (−HS). Ten virgin females were mated with 30 *CS* males and a percentage of females with an egg in the oviduct or uterus represents one data point. The bottom panel shows wild-type *CS* (OAMB++) and the *oamb* mutant females (OAMB−) carrying various transgenes (HS-GAL4, UAS-K3, UAS-ASII and UAS-ASIII) under test. UAS-ASII and UAS-ASIII are independent transgenic lines of UAS-AS. The ovulation levels of the *oamb* females expressing K3 or AS driven by HS-GAL4 were similar to that of *CS* females (OAMB, ++) but significantly different from those of the *oamb* females carrying HS-GAL4, UAS-K3, or UAS-AS alone or no transgene (ANOVA, *F*_7,67_ = 22.82, *p*<0.0001; ns, *p*>0.05 by *post hoc* Tukey Kramer tests, *n* = 3–12). Without heat shock, the *oamb* females carrying HS-GAL4 and UAS-AS had ovulation levels similar to that of *CS* females, but the ovulation levels of other lines were significantly different from that of *CS* (*F*_7,50_ = 23.94, *p*<0.0001; ns, *p*>0.05 by Tukey Kramer; *n* = 3–9). ns, not significant.

We have previously shown that females lacking both K3 and AS OAMB isoforms are defective in ovulation [9]. To investigate whether K3 or AS or both are crucial for ovulation, we conducted a rescue experiment using the GAL4/UAS binary system, in which the transcription factor GAL4 binds to UAS to activate the downstream gene expression [19]. We employed a HS-GAL4 driver, in which GAL4 expression is controlled by the heat inducible *hsp70* promoter [20], for ubiquitous expression of transgenic OAMB in *oamb* null mutants [9]. The transgenic *oamb* females carrying HS-GAL4 along with UAS-K3 or UAS-AS were subjected to heat shock at 37°C for 1 h (Figure 1, +HS) then allowed to mate with wild-type *Canton S* (*CS*) males at room temperature. To monitor ovulation status, the females were subsequently examined for the presence of an egg in the oviduct or uterus. Another groups of females were tested without heat shock (Figure 1, −HS). The *oamb* females carrying HS-GAL4, UAS-K3, or UAS-AS alone and *CS* females were tested with and without heat shock as controls. The *oamb* females with heat-induced K3 or AS expression showed ovulation levels similar to those of *CS* females. While the *oamb* females with HS-GAL4 and UAS-K3 exhibited a heat-shock dependent increase in ovulation, those with HS-GAL4 and UAS-AS showed increased ovulation in the absence of heat shock. This might be due to leaky GAL4 expression since two independent UAS-AS lines with the transgene insertion at different chromosomal locations produced similar results. The *oamb* females with HS-GAL4, UAS-K3 or UAS-AS alone did not show significant ovulation in the presence or absence of heat shock, indicating that transgene insertions or heat treatments per se do not cause enhanced ovulation. These data demonstrate that both K3 and AS are capable of reinstating normal ovulation in *oamb* females and suggest that OAMB expressed at the adult stage plays a significant role for this activity.

### OAMB is not required in the nervous system for ovulation

**Figure 2:**
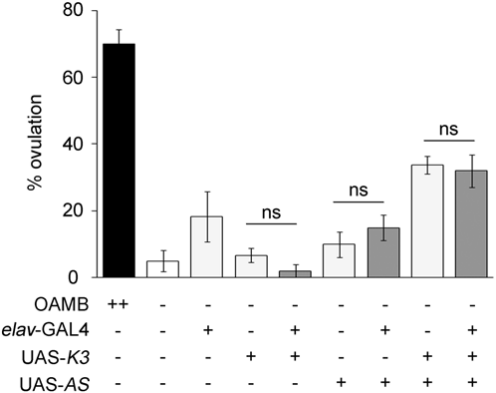
Failed rescue of the *oamb*'s phenotype with neuronal OAMB expression. Pan neuronal elav-GAL4 was used to induce K3 or AS expression in *oamb* mutant females. The ovulation levels of all *oamb* females with or without transgenes were significantly different from that of *CS* females (*F*_8,46_ = 23.42, *p*<0.0001; *n* = 5–8) and the ovulation levels of the *oamb* females carrying UAS-OAMB alone or UAS-OAMB and elav-GAL4 were comparable (ns, *p*>0.05 by Student *t*-tests).

Endogenous OAMB is expressed in the nervous and reproductive systems. HS-GAL4 is ubiquitously expressed upon heat shock and thus activates transgenic OAMB expression in all cells. To direct transgenic OAMB expression in selective tissue types in *oamb* mutants, we first used elav-GAL4, in which GAL4 is expressed in all neurons [20]. The *oamb* females carrying elav-GAL4 along with UAS-K3 or UAS-AS displayed ovulation levels comparable to those of the *oamb* females without any transgene or with elav-GAL4 and UAS-OAMB alone (Figure 2). The *oamb* females carrying both UAS-OAMB isoforms showed a small yet significant increase in the ovulation level; however, it was not further increased with elav-GAL4 (Figure 2). The *oamb* females carrying either UAS-K3 or UAS-AS used in this study have a single copy of UAS-OAMB. Thus, the *oamb* females carrying both UAS-OAMB isoforms may have higher levels of GAL4-independent OAMB expression compared to those with a single copy, leading to partial rescue of defective ovulation. We also tested another neuronal driver Cha-GAL4, which is expressed in cholinergic neurons in the central and peripheral nervous systems [21]. Similar to elav-GAL4, Cha-GAL4-driven K3 or AS expression did not rescue the ovulation phenotype of *oamb* females (data not shown). Therefore, OAMB expressed in neurons is likely dispensable or insufficient for stimulating ovulation.

### OAMB is expressed in the oviduct epithelium

**Figure 3:**
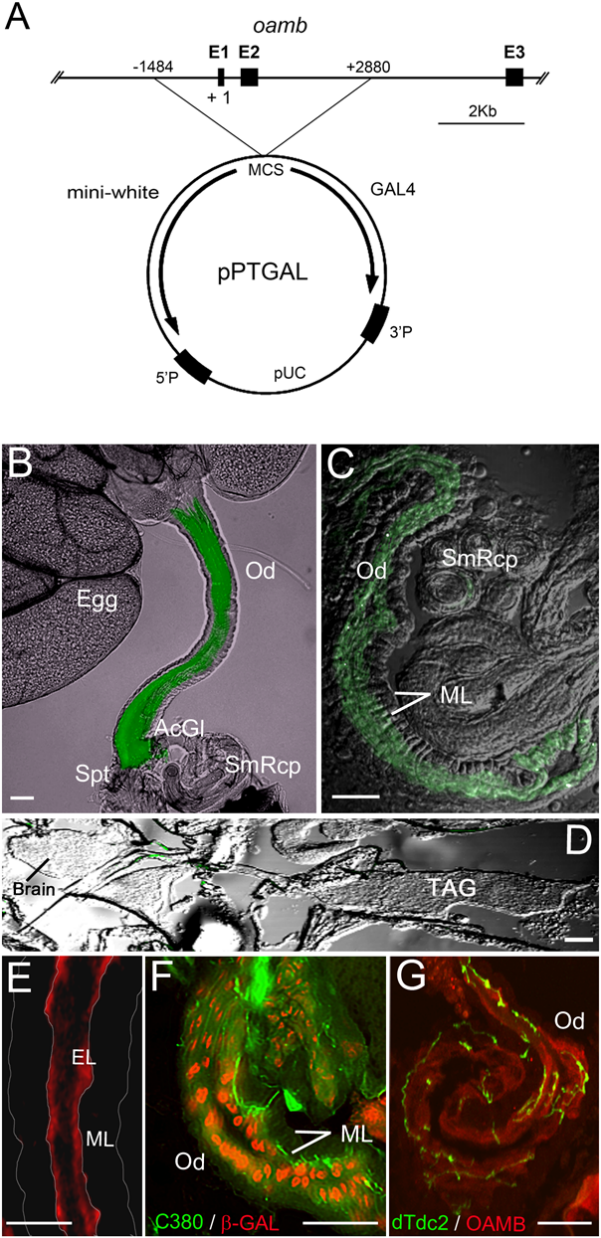
Reproductive system-specific GAL4. (A) Scheme. The 4.4 Kb *oamb* genomic region containing *oamb* promoter region and part of the second intron was cloned upstream of promoterless-GAL4 in the pPTGAL vector. E, exon; +1, transcription initiation site. (B–D) The RS-GAL4 expression pattern was visualized with *UAS-mCD8::GFP*. Strong GFP expression was detectable in the oviduct epithelium in the dissected reproduction system (B) and a sagittal section (C), but not in the central nervous system (D). Images were captured under fluorescent as well as dim light illumination to outline tissue morphologies. (E–G) Sagittal sections of the *CS* (E), *C380-GAL4/UAS-mCD8::GFP;P5391/+* (F) or *dTdc2-GAL4/UAS-mCD8::GFP* (G) female body were immunostained with anti-K3 (E, G) or anti-β-Galactosidase (F) followed by Alexa 555-labeled secondary antibodies. P5391 contains the enhancer P-element P[lArB] in the first *oamb* intron. P[lArB] has a *LacZ* reporter gene, which is expressed in the oviduct epithelial cell nuclei (F). Membrane-bound GFP induced by C380- or dTdc2-GAL4 demarcates motor or tyramine/octopamine neuronal processes, respectively, both of which project into the oviduct epithelium where OAMB-K3 immunoreactivities were evident (E, G). Od, oviduct; AcGl, accessory gland; Spt, spermatheca; SmRcp, seminal receptacle; TAG, thoracico-abdominal ganglion; ML, muscle layer. Scale bars, 50 µm. The experiments were conducted on one (F), two (G) or more than three sets (B–E) of females (5–6 females/set) generated by independent crosses, which showed consistent expression patterns of GFP, K3 and β-Galactosidase.

**Figure 4:**
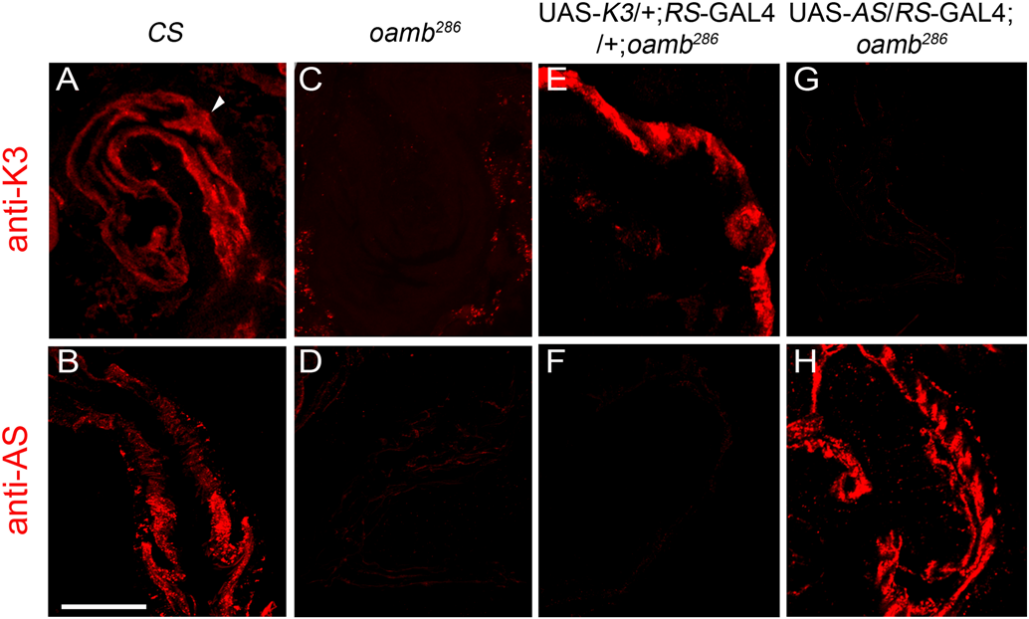
Transgenic OAMB expression driven by RS-GAL4 in the oviduct. Sagittal sections of *CS* (A, B), *oamb^286^* (C, D) and the transgenic *oamb^286^* females carrying RS-GAL4 along with UAS-K3 (E, F) or UAS-AS (G, H) were immunostained with anti-K3 (A, C, E, G) or anti-AS (B, D, F, H) followed by Alexa 555-labeled secondary antibodies. Scale bar, 50 µm. The experiments were performed on more than three sets of females (5–6 females/set) generated by independent crosses, which showed consistent K3 and AS expression patterns.

To investigate whether the major site of OAMB's function in ovulation is the reproductive system, we first explored the reproductive system-specific GAL4 drivers. We screened 67 enhancer lines selected based on the reported GAL4 expression in the reproductive system; however, none of those lines showed restricted GAL4 expression in the reproductive system. Thus, we turned our attention to the *oamb* locus for a reproductive tissue enhancer element. Our previous analysis of hypomorphic *oamb* mutants suggests that the second intron in the *oamb* gene may have a transcriptional enhancer element crucial for egg-laying [9]. We generated GAL4 enhancer lines by cloning a 4.4 Kb fragment containing this region and a putative *oamb* promoter upstream of the promoterless GAL4 gene in pPTGAL [22] (Figure 3A). The *oamb-4.4-GAL4* in all six independent lines showed prominent enhancer activities in the female reproductive system [named RS (reproductive system)-GAL4] and images of one representative line crossed with *UAS-mCD8::GFP* (membrane tethered GFP) are shown in Figure 3. Notably, the oviduct but not the nervous system showed RS-GAL4-driven GFP expression (Figures 3B–3D). The oviduct is a tubular tissue delivering mature eggs from two ovaries to the uterus and consists of two layers, circular muscles and the underlying epithelium [13]. Strong GFP expression was visible only in the epithelial cells (Figures. 3B & 3C). Consistently, immunostaining with isoform-specific OAMB antibodies revealed localization of endogenous K3 and AS proteins in the oviduct epithelium of wild-type *CS* females (Figures 3E, 4A & 4B).

The oviduct receives input from motor neurons and modulatory octopaminergic neurons in the TAG, and those neurons are shown to project to the oviduct circular muscle [10], [11]. Strong OAMB expression in the oviduct epithelium, but not the muscle, raises a question as to whether the epithelium receives input directly from the TAG neurons. To address this, we examined the transgenic females expressing membrane-tethered GFP in motor neurons via C380-GAL4 [23] or in octopaminergic neurons via dTdc2-GAL4 [11]. We also employed the *P5391* enhancer trap line containing a nuclear localization signal-tagged *LacZ* reporter gene in the first *oamb* intron [9]. In this line, β-Galactosidase encoded by *LacZ* reflects the endogenous OAMB expression pattern and thus demarcates oviduct epithelial cells. When the cryosections of the *P5391* female carrying *C380-GAL4 and UAS-mCD8::GFP* were examined for β-Galactosidase immunoreactivity and GFP, the β-Galactosidase-positive epithelial cell layer was extensively innervated by GFP-labeled motor neuronal processes (Figure 3F). Similarly, immunohistochemical analysis of the female carrying *dTdc2-GAL4* and *UAS-mCD8::GFP* with anti-K3 antibody revealed octopamine neuronal processes projecting through the muscle layer and making contacts with the OAMB-expressing epithelium (Figure 3G). As previously reported [10], [11], GFP-labeled motor and octopamine neuronal processes were also visible in close contacts with the oviduct circular muscle (data not shown). These observations suggest that the TAG motor and octopaminergic neurons directly regulate oviduct muscle and epithelial cell activities, and OAMB serves as a receptor processing the TAG octopamine input to the epithelium.

### OAMB is required in the oviduct epithelium for ovulation

We next investigated whether the *oamb* female's impaired ovulation could be rescued by restored OAMB expression in the oviduct epithelial cells. OAMB expression was monitored by immunostaining with anti-K3 and anti-AS antibodies in the transgenic *oamb* null mutants carrying RS-GAL4 and individual OAMB isoforms in which *CS* and *oamb* females served as controls. Both K3 and AS immunoreactivities were clearly visible in the *CS* oviduct epithelium (Figures 4A & 4B) but absent in the *oamb* oviduct epithelium (Figures 4C & 4D) and in the oviduct muscle and other reproductive tissues of both genotypes (Figure 4 and data not shown). Likewise, distinct isoform-specific immunoreactivities were evident in the oviduct epithelium of the transgenic *oamb* females carrying RS-GAL4 and UAS-K3 (Figures 4E & 4F) or RS-GAL4 and UAS-AS (Figures 4G & 4H). This demonstrates that RS-GAL4 effectively induces K3 and AS expression in the oviduct epithelium of *oamb* females.

**Figure 5:**
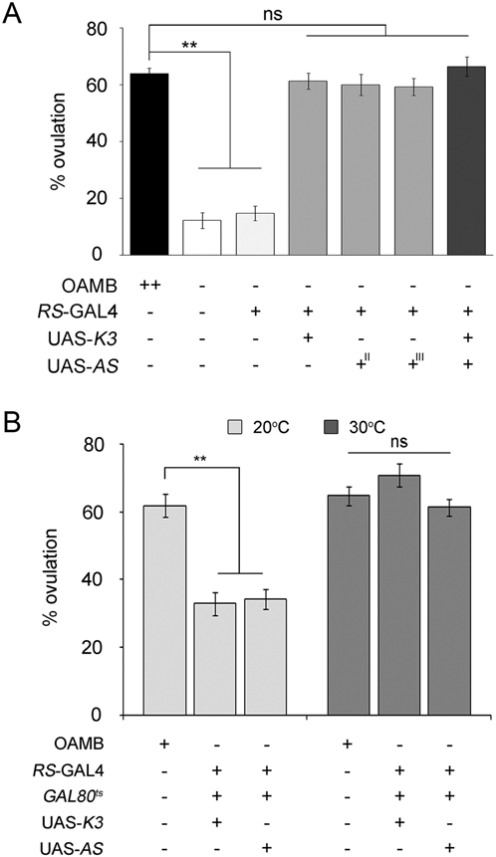
Rescue of the *oamb*'s ovulation defect by RS-GAL4-induced OAMB expression. (A) The females of various genotypes reared at 25°C were subjected to ovulation tests. The transgenic *oamb* females carrying RS-GAL4 along with UAS- K3 or UAS-AS or both show ovulation levels comparable to that of *CS* females (*F_6,89_* = 58.40; **p<0.0001 by Tukey-Kramer; ns, not significant *p*>0.05; *n* = 9–15). (B) The *oamb* females carrying GAL80^ts^ and RS-GAL4 along with UAS-K3 or UAS-AS reared at 30°C for 3 days (OAMB induction), but not at 20°C (no induction), showed similar ovulation levels to the control females (20°C, *F_2,56_* = 26.8, *p*<0.0001, *n* = 19–20; 30°C, *F_2,60_* = 2.27, *p*>0.05, *n* = 20–22).

When the transgenic *oamb* females carrying RS-GAL4 along with UAS-K3 or UAS-AS were subjected to ovulation tests, they showed ovulation levels similar to those of *CS* females (Figure 5). Interestingly, transgenic expression of both isoforms together in the *oamb* female oviduct did not further increase ovulation levels. These results indicate that OAMB expressed in the oviduct epithelium is sufficient to rescue the *oamb* female's impaired ovulation and both OAMB isoforms have similar capacities for this activity. We employed the TARGET (GAL80^ts^/GAL4/UAS) system [24] for temporally and spatially regulated transgenic OAMB expression to determine whether OAMB is required during a developmental or physiological process. In the TARGET system, GAL80^ts^ binds to GAL4 to sequester it from activating UAS. At 30°C, the temperature sensitive GAL80^ts^ no longer binds to GAL4, allowing it to act on UAS to induce the downstream gene expression and thus enabling transgene expression in a tissue- and time-dependent manner. To induce OAMB expression only in adult oviduct epithelial cells, the *oamb* females carrying tubP-GAL80^ts^ (GAL80^ts^ under the tublulin promoter for ubiquitous expression), RS-GAL4 and UAS-OAMB (either -K3 or -AS) were reared at 30°C for 3 days prior to mating. They showed ovulation levels comparable to those of *CS* females whereas ovulation levels of the *oamb* females with the same transgenes kept at 20°C remained low (Figure 5B). This indicates the significant role of OAMB during a physiological, but not developmental, process for ovulation. Taken together, the critical site for OAMB's physiological function in ovulation is the oviduct epithelium, in which transgenic expression of either isoform is sufficient to rescue the *oamb* female's phenotype.

### CaMKII is a major downstream signaling molecule of OAMB in ovulation

To delineate the cellular mechanism by which OAMB regulates ovulation, we first explored protein kinases that functionally interact with OAMB in the oviduct epithelium. OAMB activates cAMP and Ca^2+^ increases *in vitro* [16], [18]. Potential cellular molecules activated by cAMP or Ca^2+^ include protein kinase A (PKA) for cAMP and protein kinase C (PKC) and CaMKII for Ca^2+^. Since mutations in these kinases cause developmental lethality, their functions in ovulation are unknown. To assess physiological roles of PKA, PKC and CaMKII in ovulation, we employed the TARGET system with UAS-PK inhibitors or variants. A dominant negative PKAr*, which is defective in binding cAMP [25], and inhibitory peptides of PKC [PKCi; [26]] and CaMKII [ala; [27]] have been shown to effectively inhibit PKA, PKC and CaMKII activities, respectively, and thus were used to manipulate the corresponding protein kinase activities in adult oviduct epithelial cells. In an attempt to sensitize physiological effects of inhibited protein kinase activities, the experiments were conducted in the *oamb* heterozygous background.

**Figure 6:**
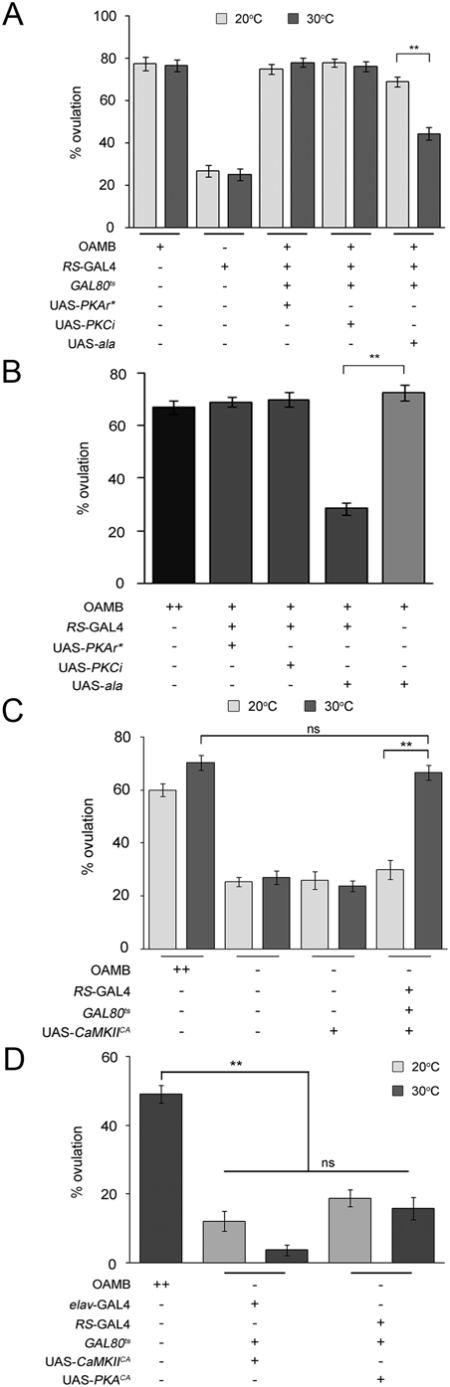
Potential protein kinases activated by OAMB in the oviduct epithelium. (A) Temporarily induced CaMKII inhibitor peptides (ala; 30°C), but not dominant negative PKA (PKA*) and PKC inhibitor peptides (PKCi), in the oviduct epithelium inhibited ovulation in the heterozygous *oamb* females (**p<0.0001 by Student *t*-test, *n* = 18–19; other pairs, *p*>0.05, n = 10–20). (B) Persistent induction of ala, but not PKA* and PKCi, driven by RS-GAL4 inhibited ovulation (ANOVA: *F_4,98_* = 53.5, ** *p*<0.0001 by Tukey-Kramer, *n* = 20–21). (C) The transgenic *oamb* females carrying RS-GAL4, GAL80^ts^ and UAS-CaMKII^CA^ had the ovulation level comparable to that of *CS* females after temperature shift (ns, *p*>0.05 by Student *t*-test), indicating full rescue of the *oamb*'s impaired ovulation by constitutively active CaMKII induced in the oviduct epithelium. *n* = 21–23, ** *p*<0.0001 by Student *t*-test. (D) The transgenic *oamb* females with neuronal CaMKII^CA^ or oviduct epithelial PKA^CA^ induced by elav-GAL4 or RS-GAL4, respectively, exhibited ovulation levels significantly lower than that of *CS* females (*F_4,75_* = 36.8, ** *p*<0.0001, *post hoc* Tukey Kramer tests; *n* = 11 for elav-GAL4 lines, *n* = 19–21 for the rest). Temperature shift in the transgenic *oamb* females carrying elav-GAL4, GAL80^ts^ and UAS-CaMKII^CA^ or those carrying RS-GAL4, GAL80^ts^ and UAS-PKA^CA^ had no effects on ovulation levels (ns, *p*>0.05).

The *oamb* heterozygous females carrying tubP-GAL80^ts^ and RS-GAL4 along with UAS- PKAr*, UAS-PKCi or UAS-ala showed normal ovulation when reared at permissive temperature 20°C (Figure 6A). However, when they were raised at 30°C for 3 days to inhibit the targeted protein kinase activity, the females with the CaMKII inhibitory peptide ala, but not PKAr* or PKCi, showed significantly decreased ovulation levels (Figure 6A). We also examined the females without GAL80^ts^, which may have increased inhibitor expression and thus enhanced protein kinase inhibition. Similar to temporal induction, the CaMKII inhibitory peptide ala constantly induced with RS-GAL4 inhibited ovulation; however, persistent induction of PKAr* or PKCi expression had no effect (Figure 6B). These results suggest that CaMKII, but not PKA or PKC, is involved in a physiological process for ovulation and may function as a downstream effector of OAMB. This is consistent with the aforementioned observations that the *oamb* female's impaired ovulation was rescued by both OAMB isoforms, whose common intracellular signal is Ca^2+^.

If CaMKII indeed functions downstream of OAMB, we reasoned that ligand-independent activation of CaMKII would bypass OAMB for stimulating ovulation and thus rescue the *oamb*'s ovulation phenotype. To test this, we used constitutively active CaMKII (CaMKII^CA^) whose activity is independent of the upstream signals Ca^2+^ and calmodulin [28]. The *oamb* homozygous females carrying tubP-GAL80^ts^, RS-GAL4 and UAS-CaMKII^CA^ were impaired in ovulation similar to *oamb* females when reared at 20°C (uninduced CaMKII^CA^); however, they exhibited ovulation levels comparable to those of *CS* females upon temperature shift (induced CaMKII^CA^; Figure 6C). On the contrary, the *oamb* females carrying tubP-GAL80^ts^, elav-GAL4 and UAS-CaMKII^CA^ or those carrying tubP-GAL80^ts^, RS-GAL4 and UAS-constitutively active PKA [PKA^CA^, [29]] did not exhibit enhanced ovulation when subjected to the same temperature shift (Figure 6D). Therefore, constitutively active CaMKII, but not PKA, expressed in the adult oviduct epithelium was sufficient to reinstate ovulation in *oamb* females whereas constitutively active CaMKII induced in the nervous system failed to rescue the ovulation defect, corroborating the unsuccessful rescue with neuronal OAMB expression (Figure 2). These data point to CaMKII as a major downstream signaling molecule that is activated by OAMB in the oviduct epithelium for ovulation. If OAMB or CaMKII represent the sole ovulation signal activated by mating, CaMKII^CA^ would induce ovulation in the absence of copulation. However, the virgin *oamb* females expressing CaMKII^CA^ in the oviduct epithelium showed negligible ovulation (data not shown). This suggests additional factors or processes mediating a mating signal, which may include those acting in the ovary or oviduct muscle.

## Discussion

Octopamine, as a major neurotransmitter, neuromodulator and neurohormone, regulates diverse physiological processes in invertebrates that include sensory information processing, egg-laying, fight or flight responses, and complex neural functions such as learning and memory [30]. These astonishingly diverse effects of octopamine are initiated by the binding of octopamine to G-protein-coupled receptors expressed in distinct tissue or cell types; however, very little is known about relevant octopamine receptors and underlying cellular mechanisms that mediate octopamine's physiological functions. In this report, we have shown that OAMB regulates ovulation in the oviduct epithelium and recruits CaMKII for this function. This role of OAMB is physiological, as opposed to developmental, since restored OAMB expression in the oviduct epithelium at the adult stage is sufficient for reinstating ovulation in *oamb* females. This is consistent with the findings observed in the octopamine-less *dTdc2* and *tβh* females, in which feeding octopamine only at the adult stage rescues the sterility phenotype of both mutants [11], [31].

Sex peptides transferred to the female during copulation enhance egg-laying upon binding to the receptor SPR expressed in the Fruitless neurons [8]. The mechanism by which the Fruitless neurons stimulate egg-laying is unknown; however, octopaminergic neurons in the TAG likely represent a downstream target since the egg-laying phenotype of *tβh* females is rescued by restored TβH expression in these neurons [10]. The TAG octopaminergic neurons project axons to various areas in the reproductive track including the ovary, lateral and common oviducts, sperm storage organs and the uterus [12], [13]. Mating induces distinctive changes in vesicle release at the nerve terminals in different areas of the reproductive track [32], some of which may represent the TAG octopamine neuronal activities. In the dissected reproductive system, octopamine application augments the amplitude of myogenic contractions of the peritoneal sheath in the ovary while it inhibits stimulated muscle contractions of the oviduct [12], [13]. These opposite effects of octopamine may be crucial for coordinated constriction and relaxation of the ovary and oviduct, respectively, in transferring a mature egg to the uterus. OAMB may serve as a receptor processing the octopamine's input in the oviduct while another octopamine receptor may mediate the constriction signal in the ovarian peritoneal sheath, which lacks OAMB expression.

**Figure 7:**
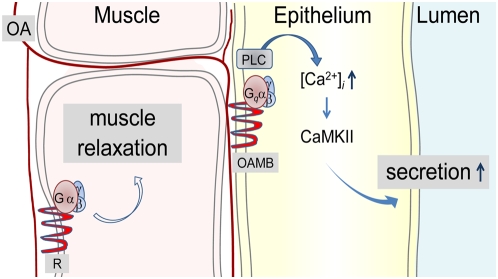
Working model for OAMB's roles in the oviduct epithelium. Mating activates the TAG octopaminergic neurons projecting to the oviduct epithelium where OAMB, upon receiving octopamine input, stimulates fluid secretion via signal transduction pathways involving CaMKII for egg transport from the ovary to the uterus. In addition, the octopamine input may be processed by another octopamine receptor in the oviduct muscle for relaxation. Concerted activities of an epithelial OAMB and a muscle receptor may be crucial for egg transfer through the oviduct.

Remarkably, OAMB's activity is required in the epithelium rather than the muscle for normal ovulation. Consistent with this, the histochemical analysis reported here reveals extensive innervation of the TAG octopamine neuronal processes into the oviduct epithelial layer where both OAMB isoforms are enriched, in addition to the muscle. This raises an important question regarding the nature of an OAMB's role in ovulation. While no information is available on the oviduct epithelium in *Drosophila* or other insects, studies of the mammalian oviduct indicate active roles of the epithelium in fluid secretion and ciliary activity for gamete and embryo transport [33]. Similarly, it is possible that OAMB in the *Drosophila* oviduct epithelium is involved in regulating fluid secretion to establish proper luminal environment and possibly ciliary action for egg transport. The capacity of either OAMB-K3 or OAMB-AS to reinstate ovulation in *oamb* females strongly implicates intracellular Ca^2+^ rather than cAMP as a downstream effector. This is corroborated by our findings demonstrating CaMKII as a key epithelial component downstream of OAMB. It is uncertain whether individual isoforms or two isoforms together have comparable efficacies in activating CaMKII and ovulation. Future studies employing quantitative manipulation of transgenic OAMB expression may clarify this issue. Taken together, the epithelial OAMB stimulated upon mating likely activates CaMKII via increased intracellular Ca^2+^, which may in turn trigger biochemical changes necessary for fluid secretion (Figure 7). Potential molecules involved in this process may include transporters, ion channels, Na^+^-K^+^-ATPase and the molecules involved in cilia movements [33]. In the absence of OAMB, epithelial cell activities and fluid may be inadequate for egg movement, leading to ovulation failure. Since octopamine induces relaxation in the dissected oviduct, relaxation may involve another octopamine receptor in the muscle, and concerted activities of OAMB and a muscle receptor may be crucial for successful egg transport (Figure 7). This working model is currently under test.

Octopamine regulates oviduct activities in other insects as well. In the locust oviduct, octopamine inhibits the basal tonus and neurally evoked muscle contractions, which are mediated by cAMP-dependent mechanisms [34], [35]. These effects of octopamine may be mediated by an OAMB-like receptor with the different intracellular effector cAMP. Alternatively, they may involve another octopamine receptor(s) present in the muscle. *Drosophila* has three octopamine receptors (OctβR1, 2 and 3) that can also stimulate cAMP increases [36]. Spatial expression patterns of three OctβRs are as yet unknown. It is conceivable that an OctβR or OctβR-like receptor, possibly present in the *Drosophila* or locust oviduct muscle, respectively, is additionally involved in ovulation by inducing muscle relaxation through a cAMP signaling pathway (Figure 7). At present, molecular components and cellular pathways controlling ovulation are largely unknown and likewise very little is known about the oviduct functions and mechanisms. Our findings reported here uncover the critical roles of the oviduct epithelium and its cellular components OAMB and CaMKII in ovulation. Future studies to identify additional downstream effectors of OAMB and their functions should help further our understanding of the important reproductive process ovulation and provide novel insights into the development of effective insecticides. Typically, intracellular signals activated by G-protein-coupled receptors are characterized in *in vitro* cell lines. This study has identified the intracellular signal activated by the G-protein-coupled receptor OAMB *in vivo* that has functional significance. Similar approaches could be applied to other receptors to investigate rather poorly defined cellular mechanisms that G-protein-coupled receptors activate for their *in vivo* functions.

Norepinephrine, a mammalian counterpart of octopamine, also plays profound roles in female reproduction by acting on the reproductive and nervous systems. Sympathetic nerve terminals containing norepinephrine innervate the ovaries, oviducts, and uterus. Moreover, norepinephrine levels in the human fallopian tube vary in a region- and estrous cycle-dependent manner being the highest in the isthmus and the fimbriated end at the time of ovulation [37]. When assayed *in vitro*, adrenergic receptor agonists not only modulate oviduct muscle activities [38], [39] but they also stimulate fluid secretion possibly via Ca^2+^-dependent mechanisms [33], [40]. Oviduct fluid in mammals is critical for egg transport, maturation and fertilization; however, the cellular process regulating its secretion is largely unknown. Damage in the oviduct epithelium is associated with pelvic inflammatory disorder, leading to hydrosalpinx formation and reduced fertility [33], [41], [42]. Thus, enhanced understanding of physiological and cellular factors and processes controlling oviduct fluid will provide significant insights into healthy reproduction as well as impaired fertility associated with pelvic inflammatory disorder and other related disorders.

## Materials and Methods

### Drosophila strains and culture

The *oamb* mutants used in this study are the null alleles *oamb^286^* and *oamb^96^* [9]. The transgenic lines *C380-GAL4*, *dTdc2*-GAL4, *elav-GAL4*, *tubP-GAL80^ts^*, *UAS*-PKAr*, *UAS*-*PKA^CA^* (also known as UAS-mc*), *UAS-CaMKII^CA^* and *UAS-ala* were kindly provided by Drs. M. Ramaswami, J. Hirsh, M. Heisenberg, R. Davis, J. Kiger, Jr., D. Kalderon and L. Griffith, respectively. In addition, *heat shock (HS)-GAL4*, *UAS*-*PKCi*, and *UAS*-*mCD8::GFP* were obtained from the Bloomington Stock Center. All fly stocks were reared on standard cornmeal/sugar/yeast medium at 25°C, unless otherwise stated, with approximately 50% relative humidity under a 12 h light/12 h dark cycle.

### UAS-OAMB cDNA and RS-GAL4 constructs

For transgenic expression of OAMB, K3 [16] or AS cDNA [9] was cloned under UAS in pPBretU vector [43]. Four independent transgenic lines *UAS-K3-I*, *UAS-K3-III*, *UAS-AS-II*, and *UAS-AS-III* were established by germ line transformation in the *ry^506^* genetic background and the transgene insertion sites were identified by inverse PCR and sequencing [20]. To generate transgenic lines expressing GAL4 in the oviduct, the endogenous *oamb* regulatory region (−1484 to +2880) was amplified by PCR using primers 5′-CACTAGTCCACGTCAGCCCATTC-3′ and 5′-GAAGATCTCCCTGTGCTTGGC-3′, cloned in the pPTGAL vector containing the promoterless GAL4 gene [22], and germ-line transformed in the isogenic *w^1118^* to generate six independent lines. The GAL4 expression patterns were visualized after crossing with *UAS-mCD8::GFP*. Since all six lines showed similar expression patterns, one line (*RS-GAL4*) with the GAL4 insertion on the second chromosome was used for all experiments described here after backcrossed with the isogenic *w^1118^* for six generations. The individual transgenes (HS-GAL4, elav-GAL4, RS-GAL4, UAS-OAMB, and UAS-CaMKII^CA^) were placed in the *oamb^286^ or oamb^96^* null mutant background for rescue experiments.

### Immunohistochemistry

A 315 bp fragment of AS cDNA corresponding to the putative third intracellular loop specific to the AS isoform was amplified by PCR using 5′-TGAATTCGCATACATAGAGGACGA-3′ and 5′-TCTCGAGCGTGCTGCTGCTGCTGT-3′ primers and cloned in pGEX-4T-2 (Pharmacia, Piscataway, NJ) to generate a fusion protein with glutathione *S*-transferase. As previously described [16], a 542 bp fragment corresponding to the putative third intracellular loop of the K3 isoform was also used to generate the fusion protein. After the third injection of the fusion proteins into Swiss Webster mice, ascites fluid was collected and used for immunostaining. Flies were fixed in 4% paraformaldehyde and 40 mM lysine in phosphate buffered saline, pH 7.2, for 3 h at 4°C and then kept in 25% sucrose overnight. Ten-micron sagittal cryosections of the whole fly were subjected to immunostaining as previously described [44] using rabbit anti-β-Galactosidase antibody (1∶1000, Cappel Lab., Cochranville, PA), mouse anti-K3 antibody (1∶200), mouse anti-AS antibody (1∶50), and Alexa 555-conjugated anti-rabbit or anti-mouse IgG antibody (1∶1000; Invitrogen, Eugene, OR) [9]. The antigen specificities of anti-K3 and anti-AS antibodies were established based on deficient K3 and AS immunoreactivities in *oamb^286^* null mutants and negligible cross-reactivities in the *oamb* mutants expressing transgenic K3 or AS isoform as shown in the results section. Images were acquired with the DMR epifluorescent (Leica, Heidelberg, Germany) or FluoView confocal (Olympus, Melville, NY) microscope. All chemicals were purchased from Sigma-Aldrich (St. Louis, MO) and Fisher Science (Fair Lawn, NJ).

### Ovulation test

Virgin females were collected within 12 h after eclosion. Ten 5 to 7 day-old females were housed with 30 wild-type *CS* males in a food vial for 3 h for mating and then the female reproductive system was dissected to examine the presence of an egg in the oviduct or uterus. The percentage of females with an egg per vial was used to represent one data point. To induce transgenic *oamb* gene expression at the adult stage, the *oamb* mutant females carrying HS-GAL4 along with UAS-K3 or UAS-AS reared at 18° to 20°C were treated with heat shock at 37°C for 1 h twice a day for 3 days and then tested for ovulation. For the experiments involving tubP-GAL80^ts^, 2 to 3 day-old virgin females were reared at 30°C for 3 days and then subjected to mating and ovulation tests at room temperature. The females kept at 20°C were used as an uninduced control. Statistical analyses were performed using Minitab Release 14.1 (Minitab, State College, PA). All data are presented as mean±SEM and were analyzed using ANOVA and *post hoc* Tukey–Kramer or Student's *t*-tests.
